# Draft Genome Sequence of Pseudomonas toyotomiensis KF710, a Polychlorinated Biphenyl-Degrading Bacterium Isolated from Biphenyl-Contaminated Soil

**DOI:** 10.1128/genomeA.00223-15

**Published:** 2015-04-02

**Authors:** Takahito Watanabe, Atsushi Yamazoe, Akira Hosoyama, Hidehiko Fujihara, Hikaru Suenaga, Jun Hirose, Taiki Futagami, Masatoshi Goto, Nobutada Kimura, Kensuke Furukawa

**Affiliations:** a Research Institute for Sustainable Humanosphere, Kyoto University, Uji, Kyoto, Japan; b Biological Resource Center, National Institute of Technology and Evaluation (NITE), Shibuya-ku, Tokyo, Japan; c Department of Food and Fermentation, Faculty of Food and Nutrition, Beppu University, Beppu, Oita, Japan; d Bioproduction Research Institute, National Institute of Advanced Industrial Science and Technology (AIST), Tsukuba, Ibaraki, Japan; e Department of Applied Chemistry, Faculty of Engineering, University of Miyazaki, Miyazaki, Japan; f Education and Research Center for Fermentation Studies, Faculty of Agriculture, Kagoshima University, Kagoshima, Japan; g Laboratory of Future Creation Microbiology, Faculty of Agriculture, Kyushu University, Fukuoka, Japan

## Abstract

Pseudomonas toyotomiensis KF710 utilizes biphenyl and degrades polychlorinated biphenyls (PCBs). Here, we report the genome sequence of the KF710 strain, consisting of 5,596,721 bp with 5,155 coding sequences. The biphenyl catabolic genes were almost identical to those of Pseudomonas pseudoalcaligenes KF707, one of the most well-characterized biphenyl-utilizing strains.

## GENOME ANNOUNCEMENT

Pseudomonas toyotomiensis KF710 (NBRC 110674) was one of the 14 biphenyl-utilizing bacteria, termed KF strains, which were isolated from biphenyl-contaminated soil in Kitakyushu, Japan (1). This strain grows on biphenyl and degrades polychlorinated biphenyls (PCBs), which are known to be serious environmental pollutants. We previously reported that the Southern hybridization profiles of the biphenyl-catabolic (*bph*) genes exhibited by the KF710 strain and some KF strains are similar to those exhibited by Pseudomonas pseudoalcaligenes KF707, one of the most well-characterized biphenyl-utilizing strains (1–3). The high similarities between the *bph* genes are of interest. Therefore, the genomic information of these strains will provide insights into the diversity and molecular relationships of these biphenyl-utilizing bacteria.

A whole-genome shotgun approach was utilized for the KF710 strain using a combined method of shotgun sequencing on a Roche 454 GS FLX+ system and paired-end sequencing on a HiSeq sequencing system (Illumina). The Newbler software package (version 2.6; Roche) was used for the genome assembly. The draft genome size was 5,596,721 bp, containing 29 contigs with an average contig length of 192,990 bp, a median coverage depth of 115-fold, and an average G+C content of 62.6 mol%.

The genome sequence was uploaded to the RAST server (4). A comparison of the KF710 strain with other bacteria within the RAST server identified Pseudomonas mendocina ymp (genome ID 399739.6) (5) as its closest neighbor, with a score of 542, followed by the KF707 strain (genome ID 1149133.6), with a score of 511. Rapid genome annotation using the RAST annotation server (4) described 5,155 coding sequences (CDSs) and 62 structural RNAs. The CDSs were classified into 520 subsystems. The subsystem feature count of the metabolism of aromatic compounds in the KF710 strain (*n* = 167 CDSs) was more than three times that in the ymp strain (*n* = 49) but less than that in the KF707 strain (genome ID 1149133.11) (*n* = 223). These differences may reflect the different capacities for gene transfer through the mobile genetic elements carrying catabolic genes for various aromatic compounds (6).

A detailed comparison revealed that the KF710 *bph* genes, consisting of *bphA1A2(orf3)bphA3A4BCX0X1X2X3D*, were >99.5% identical to those of the KF707 strain (7). Several mobile element proteins were located in the proximal regions of the *bph* genes in the KF710 strain, as well as in the KF707 strain. However, the genes outside these regions were different. These findings suggest that the *bph* genes of the KF710 strain were transferred through the mobile genetic elements and integrated into the chromosome. We previously reported that an approximately 90-kb DNA region containing the *bph* and salicylate (*sal*)-catabolic genes in Pseudomonas putida KF715, termed the *bph-sal* element, can be transferred by conjugation to other P. putida strains (8). Therefore, the comparative genomics of the KF strains will provide a better understanding of how such catabolic genes for aromatic compounds behave in the natural environment.

### Nucleotide sequence accession numbers.

The nucleotide sequence of P. toyotomiensis KF710 has been deposited in the DDBJ/EMBL/GenBank databases under the accession numbers BBQO01000001 to BBQO01000029.
